# Cutaneous Coccidioidomycosis and Basal Cell Carcinoma: Case Report on a Diagnostic Dilemma

**DOI:** 10.7759/cureus.43374

**Published:** 2023-08-12

**Authors:** Prajwala Nagarajappa, Maneeth Mylavarapu, Srivathsava Gurumurthy, Jaime A Tschen

**Affiliations:** 1 Internal Medicine, Mysore Medical College and Research Institute, Mysore, IND; 2 Public Health, Adelphi University, Garden City, USA; 3 Dermatopathology, St. Joseph Dermatopathology, Houston, USA

**Keywords:** erythema nodosum, immunocompromised, breast cancer, basal cell carcinoma, coccidioidomycosis

## Abstract

In the world of medical diagnoses, a particularly intriguing scenario unfolds, wherein the cutaneous manifestation of a systemic fungal infection disguises itself as a Basal Cell Carcinoma (BCC), a skin cancer. Coccidioidomycosis is an endemic fungal infection caused by inhaling spores of the fungus Coccidioides immitis. It is primarily a pneumonic illness which, in a few cases, has the potential to cause severe systemic disease. In this article, we report a case of a 60-year-old female with a known history of infiltrating ductal carcinoma of left breast status post lumpectomy and adjunct chemotherapy presented with complaints of cough, fever, and easy fatigability that seemed to be attributable to her immunocompromised state. She also had a pseudo-vesicular plaque on her left upper arm for two years. As we delve into the case, it becomes clear that Coccidioidomycosis and other opportunistic infections are commonplace among immunocompromised patients. Prior awareness of this condition and a cautious yet open-minded approach prevented gross misdiagnosis in our case. Physicians should be vigilant in diagnosing Coccidioidomycosis, especially in immunocompromised patients presenting with mild constitutional symptoms in endemic regions. Early detection and management are crucial to prevent severe complications and increase patient survival rates.

## Introduction

Coccidioidomycosis, or San Joaquin Valley fever, is an invasive disease caused by the dimorphic fungus *Coccidioides immitis*. It is primarily a pneumonic illness that, in a few cases, can potentially cause severe systemic disease [1]. It is endemic to arid regions in the southwestern US, some parts of Mexico, Central and South America. In 2019, there were 20,003 cases of Valley fever reported to the CDC, the majority of them from Arizona and California. The incidence rates are typically highest among people aged 60 and older [2]. The infection is contracted by the inhalation of arthroconidia, which later become spherules inside the lung and tissues. These spherules rupture, releasing endospores phagocytosed by macrophages and T cells. This results in an asymptomatic subclinical infection in 60-70% of individuals.

Coccidioidomycosis is a leading cause of community-acquired pneumonia in highly endemic areas, accounting for nearly 15% to 30% of cases in some areas within the endemic region. Fever, cough, shortness of breath (SOB), and chest pain are the most frequently encountered complaints. Unilateral infiltrates on X-ray are sometimes accompanied by lung cavities. Hilar and paratracheal adenopathy suggests an extrathoracic spread of the disease [3]. It can disseminate to extrapulmonary locations (0.5-2% of cases) and is more common in people with impaired cell-mediated immunity. In such cases, skin lesions are common, which include a transient, faint, maculopapular rash early in the disease, erythema nodosum, or erythema multiforme. Joint involvement is seen, with knee joints being the commonest. In 90% of cases, central nervous system (CNS) involvement is seen, which is fatal if left untreated [4].

## Case presentation

A 60-year-old female hailing from the southwestern region of the United States came for a regular follow-up with complaints of fever, cough, and easy fatigability. She also had a rash on her left upper arm for two years, which was previously undiagnosed. Past history included left-sided breast carcinoma with biopsy revealing T1N0M0, poorly differentiated, infiltrating ductal carcinoma with a high nuclear grade, and sentinel lymph nodes negative for metastasis. She was hormone receptor-positive (ER 3+, PR 3+), HER2-Neu negative, with significant proliferation (Ki67 - 15% positive). This was managed with lumpectomy followed by four cycles of adjuvant chemotherapy with Adriamycin and Cytoxan. Later she was on hormonal therapy, Tamoxifen, for the past five years. Her BRCA1 and BRCA2 were negative by MyRisk genetic test analysis.

**Figure 1:**
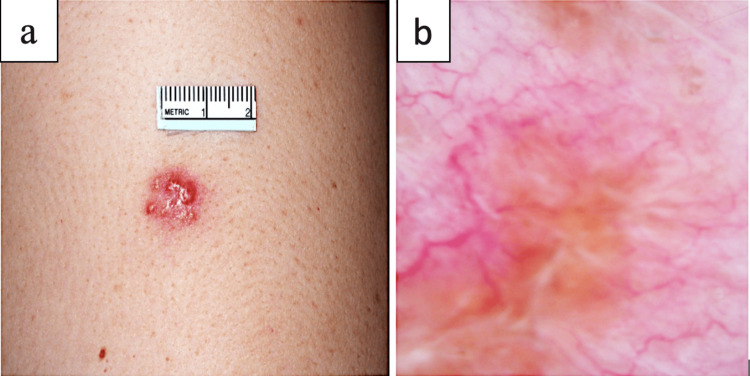
Gross findings of the rash over the left upper arm a) Pseudo vesicular plaque measuring 1.2cm x 1cm with overlying erythema b) Dermatoscopy of the lesion showing telangiectatic vessels

Local examination of the rash revealed a 1.2cm pseudo-vesicular plaque over the left deltoid region, with overlying erythema and telangiectasia. Gross and dermatoscopic findings are described in Figure 1. On examination of lymph nodes, significant lymphadenopathy was seen. Other systems examinations were normal. Her systemic symptoms were attributed to her immunocompromised state and were treated conservatively. She was also referred to a dermatologist for a shave biopsy of the rash.

**Figure 2:**
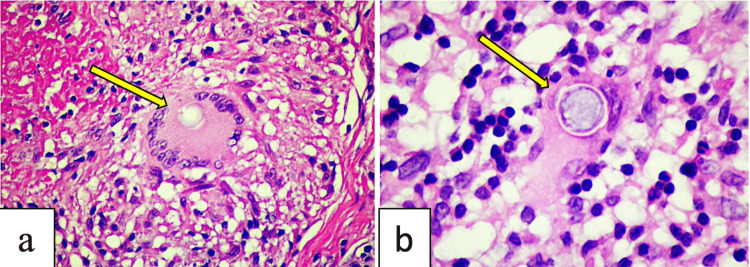
Hematoxylin and Eosin (H&E) stained section of the lesion a) Magnification of 200 showing a multinucleated giant cell with endospore-containing spherules b) Higher magnification (400) showing the encapsulated endospore with spherules

**Figure 3:**
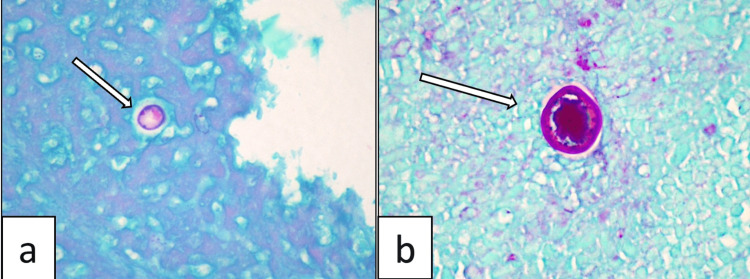
Special stains for fungus: Periodic acid-Schiff (PAS) a) Magnification of 200 showing the capsule of the endospore (PAS positive) b) Higher magnification (400) showing the encapsulated endospore

Histopathology of the shave biopsy of the lesion revealed several scattered granulomas in the dermis. These included lymphocytes, histiocytes, and endospore-containing spherules, some of which are seen within the giant cells that phagocytosed them (Figure 2). Polarizing microscopy was negative for birefringence. Special stains (Figure 3) with Periodic Acid Schiff (PAS) stain confirmed the presence of Coccidioidomycosis. Acid-Fast Bacillus stain was negative.

The patient was referred for a CT chest, which revealed a lung nodule measuring 0.9 cm in the right middle lobe which suggested disseminated Coccidioidomycosis involving skin, lungs, and lymph nodes. Conservative management with Fluconazole was initiated, upon which the patient's symptoms improved, and six months later, a repeat chest CT showed no progress in the size of the lesion. The findings of these investigations have been summarized in Table 1.

**Table 1:** Histopathology and Imaging Findings AFB, Acid-Fast Bacilli; CT, Computerized Tomography; PAS, Periodic acid-Schiff

S.No.	Investigation	Results
1.	Shave biopsy of the lesion	
	Gross examination	1.2 cm x 1 cm measuring pseudo vesicular plaque
	Microscopy	Scattered granulomas with endospore-containing spherules, the largest showing central necrosis. Polarizing microscopy negative for bi-refringence
2.	Special stains	PAS stain confirmed the presence of Coccidioidomycosis. AFB stain was negative.
3.	Chest CT	A lung nodule measuring 0.9 cm in the right middle lobe
4.	Chest CT (6-month follow-up)	Stable nodule
5.	Annual Mammogram	No signs of reoccurrence

## Discussion

Cutaneous manifestations in Coccidioidomycosis are usually reactive. They include erythema nodosum, erythema multiforme, reactive interstitial granulomatous dermatitis, acute generalized exanthema, and Sweet's syndrome. Erythema nodosum is the most common cutaneous manifestation of Coccidioidomycosis. On histological examination, they contain septal fibrosis, thickened vessels with mononuclear cells, and focal granulomatous inflammation. Occasionally, multinucleated giant cells could also be seen as found in our patient. Erythema multiforme is another common manifestation characterized by target-like lesions, possibly due to a hypersensitivity response to the coccidioidomycosis infection. Interstitial granulomatous dermatitis as a reactive cutaneous manifestation due to Coccidioidomycosis usually presents as edematous, indurated cutaneous papules, nodules, and plaques, and on histological examination, macrophages, eosinophils, neutrophils, and leukocytoclastic debris are seen. Reactive manifestations usually do not contain visible microorganisms and occur during acute primary pulmonary infection in most patients (50%) [5].

Similarities of the lesions on gross examination and the absence of the organisms in the lesions make it tough to differentiate between lesions of Coccidioidomycosis and cutaneous tumors or other granulomatous diseases. As we have seen in this case, Coccidioidomycosis, caused by the fungus Coccidioides species, can manifest in the skin and could have been mistaken for localized skin cancer, 'Basal Cell Carcinoma.' Shah et al. reported a similar situation where extensive evaluation for malignancy and other granulomatous diseases was done before getting a fungal culture. This ultimately led to the correct but delayed diagnosis of Coccidioidomycosis [6]. Thorough medical history, physical examination, and appropriate diagnostic tests are essential in differentiating between the potential causes. Early detection and management of Coccidioidomycosis are crucial to prevent severe complications, and physicians should consider rare opportunistic infections in their differential diagnosis for such patients.

## Conclusions

Diagnostic dilemmas are one of the leading causes of misinterpretation of patient presentation. In this example, a systematically disseminated fungal infection with high case fatality rates could be perceived as an easily excisable locally spreading basal cell carcinoma (BCC). Hence a high degree of suspicion is required in immunocompromised patients towards diagnosing opportunistic infections. In conclusion, cutaneous manifestations of Coccidioidomycosis, being rare, should be a differential up every physician's sleeve, particularly in immunocompromised patients.
